# Initial dosimetric experience with mega voltage computed tomography detectors and estimation of pre and post-repair dosimetric parameters of a first Helical Hi-Art II tomotherapy machine in India

**DOI:** 10.4103/0971-6203.51933

**Published:** 2009

**Authors:** Rajesh A. Kinhikar, Zubin Master, Dipak S. Dhote, Deepak D. Deshpande

**Affiliations:** Department of Medical Physics, Tata Memorial Centre, Parel, Mumbai 400012, India; 1 Brijlal Biyani Science College, Amravati, Maharashtra, India

**Keywords:** Tomotherapy, mega voltage computed tomography detector, Dosimetric stability

## Abstract

A Helical Tomotherapy™ (HT) Hi-Art II (TomoTherapy, Inc., Madison, WI, USA) has been one of the important innovations to help deliver IMRT with image guidance. On-board, mega voltage computed tomography (MVCT) detectors are used for imaging and dosimetric purpose. The two objectives of this study are: (i) To estimate the dosimetric and general capability (TomoImage registration, reconstruction, contrast and spatial resolution, artifacts-free image and dose in TomoImage) of on-board MVCT detectors. (ii) To measure the dosimetric parameters (output and energy) following major repair. The MVCT detectors also estimated the rotational output constancy well. During this study, dosimetric tests were repeated after replacing MVCT detectors and the target. fixed-gantry/fixed-couch measurements were measured daily to investigate; the system stability. Thermoluminescense dosimeter (TLD) was used during both the measurements subsequently. The MVCT image quality with old and new detectors was comparable and hence acceptable clinically. The spatial resolution was optimal and the dose during TomoImage was 2 cGy (well within the manufacturer tolerance of 4 cGy). The results of lateral beam profiles showed an excellent agreement between the two normalized plots. The output from the rotational procedure revealed 99.7% while the energy was consistent over a period of twelve months. The Hi-Art II system has maintained its calibration to within +/− 2% and energy to within +/− 1.5% over the initial twelve-month period. Based on the periodic measurements for rotational output and consistency in the lateral beam profile shape, the on-board detector proved to be a viable dosimetric quality assurance tool for IMRT with Tomotherapy. Tomotherapy was stable from the dosimetric point of view during the twelve-month period.

## Introduction

Intensity modulated radiotherapy (IMRT) has been a major paradigm shift in cancer management and its clinical application is still evolving. A Helical Tomotherapy™ (HT) Hi-Art II (TomoTherapy, Inc., Madison, WI, USA) has been one of the important innovations to deliver IMRT with image guidance.[1–4] On-board, mega voltage computed tomography (MVCT) Xenon-based detectors are used for imaging and dosimetric purposes. A 6 MV linear accelerator (LINAC) is mounted on a ring gantry which rotates continuously while the treatment couch is translated along the axis of gantry rotation during treatment delivery. A 64 leaf binary collimator is used to subdivide the fan beam into beamlets. Intensity modulation (IM) is thus achieved by a temporal modulation of the collimator leaves. MVCT detectors are mounted opposite to the LINAC.

In July, 2007, an HT machine was installed at the Advanced Centre for Treatment Research and Education in Cancer (ACTREC), Navi Mumbai, making it the first machine in India. The structural details of the machine are discussed elsewhere.[5–6] The complex design of the unit requires a high-end mechanical control and extreme synchronization to modulate radiation beam intensity.[5–6] Thus an extensive mechanical and dosimetric verification is required.[7–8]

An HT machine needs continuous monitoring due to daily fluctuations in output and hence the energy. Very often, the ion chambers are used to measure the output in static mode. Same is the case for profile measurements in water tank. MVCT detectors are used to measure the rotational output and beam profile shape. The data collected by the MVCT detectors is signature data.

The entire dosimetry equipment (ion chambers, electrometers, water tank and phantoms) is supplied by Tomotherapy Inc. The nominal dose rate of the machine is 890 cGy/min defined at source to axis distance (SAD) of 85 cm for a field size of 5 cm × 40 cm at a depth of 1.5 cm. The dose rate is measured daily for its consistency. Fluctuations in output and energy of the machine do not necessarily demand the dosimetry with independent dosimetry equipment. However, as part of quality assurance check, dosimetry may be verified with independent dosimetry system.

This study began with two objectives. The first was, to estimate the dosimetric and general capability (TomoImage registration, reconstruction, contrast and spatial resolution, artifacts free image and dose during TomoImage) of on-board MVCT detectors. The data collected by MVCT detectors was then compared with the ion chamber measurements in the water tank supplied by the manufacturer. The pre and post-replacement performance of the MVCT detector was compared as well.

The second objective was to monitor the dosimetric stability (output and energy) on a daily basis for a period of twelve months since commissioning, thus validating the third part dosimetry system. Thermoluminescense dosimeter (TLD) was used during both the measurements subsequently. The surface dose was estimated from the TLD measurements. The measurements with this independent dosimetry system were then compared with the measurements carried out with the dedicated dosimeters supplied by the manufacturer.

## Materials and Methods

**Figure 1:**
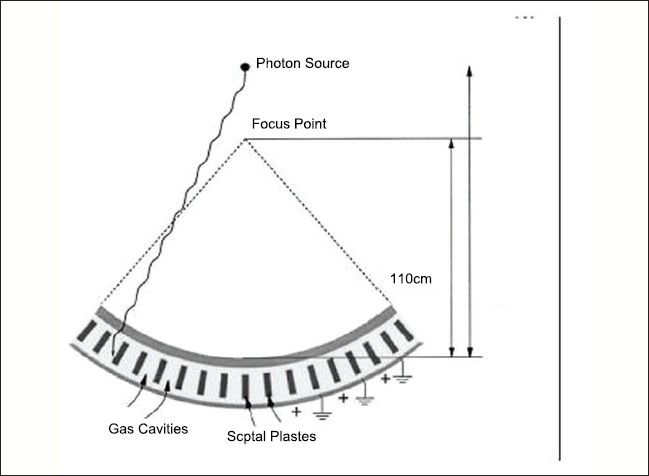
MVCT Detector

A linear array CT detector resides on the rotating gantry opposite the LINAC source [Figure 1]. This detector consists of 738 xenon-filled ion chamber detectors. Each detector has a projected transverse width of 0.73 mm at the isocenter. Each detector comprises of two gas cavities divided by thin septal plates made of tungsten. Source to Detector distance (SDD) is 142cm (57cm below isocentre of 85 cm). High-contrast resolution of 1.6 mm is achieved for 512 × 512 images.

### Dosimetric Tests

#### Tongue and Groove Procedure

This test was performed to check the primary beam alignment and stability in the X direction. The exact positioning of the radiation source is critical due to narrow beamlets and relatively short distance from the source to the primary collimator. The purpose of this test was to verify that the source is centered in the X direction with respect to the MLC, and that the source and MLC are stable as the gantry rotates. Placing the source in a position that it is aligned to the center of the MLC ensures profile symmetry consistency in the International Electro technical Consortium (IEC) X direction (crossline).

To accomplish this test, the “rotational tongue and groove procedure (TandG)” was used. Radiation was delivered (with gantry at 0°) in which every even leaf was open with all odd leaves closed, followed by every odd leaf open with all even leaves closed. This produced a series of T and G modulations. This procedure was delivered with the couch fully retracted from the bore. The on-board MVCT detector data was used to analyze the TandG results. The xenon detector recorded the incident dose profile. The profile was visually checked for its symmetric pattern. In addition, the “Net per cent out of Focus” and “Linac shift” were also calculated from the profile.

### Rotational beam stability

This test is a constancy (output and energy) quality assurance check. The purpose of this test was to evaluate the performance of the linac output and energy with the help of on-board MVCT detector array. A rotational treatment was delivered with all leaves open, a jaw width of 1 cm and the couch retracted out of the bore. The MVCT detector captures the shape of the lateral beam profile at each linac pulse. The data is averaged over the maximum number of rotations that fit in the set. The pulse by pulse Hi-Art ion chamber measurements characterize the variation of output with gantry angle. The ratio of the measured average profile to a reference profile was obtained and the constancy (output and energy) was estimated.

### Image Quality and Dose Verification

The purpose of this test was to ensure that TomoImage quality and dose is within the factory specifications. The TomoPhantom was used for this purpose. The resolution plugs with known diameter were inserted in the phantom. The ion chamber plug nearest the center of the phantom was removed and an A1SL (0.05 CC, Standard Imaging, Middleton, WI) ion chamber was inserted. A procedure with an open field rotating was performed. The procedure was performed and images were captured. It was further checked if TomoImage could be registered and viewed. The contrast (plug diameters) and spatial resolution was estimated. The charge from the electrometer was recorded and the dose was calculated. The images were checked for the resolution and the artifacts.

### Profile Measurement

**Figure 2:**
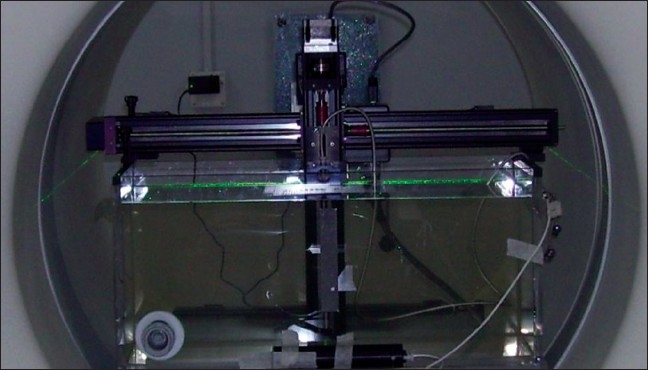
A customized water tank for profile measurement with ion chamber.

A customized water tank (Dimensions: 45 cm width, 75 cm length and 30 cm height) supplied by the manufacturer is shown in Figure 2. It was positioned on the patient table about 50 cm from the front end. This water tank has only two-dimensional movements (longer direction and the vertical). The A1SL ion chamber was placed into the water tank. The A17 (1.9 CC) ion chamber (Standard Imaging, Middleton, WI) in its buildup cap was used as stationary reference chamber and placed just outside the tank. A static procedure was selected with the field width 5 cm × 40 cm (all leaves open) and the high energy and low-energy beam lateral profiles were first measured in water at a depth of 1.5 cm. The source to surface distance (SSD) was 85 cm. An eight-channel electrometer (TomoElectrometer, Application Bind V 2.2) capable of measuring collected charge every 100 ms was used for measurements. The same high-energy and low-energy beams were measured using on-board MVCT detectors. The couch was positioned so that nothing attenuated the beam except the gantry covers. The water-tank-measured high-energy beam profile was divided by the water tank measured, low-energy beam profile and normalized to unity at the isocenter. Likewise, the onboard detector measured high-energy profile was divided by the low-energy profile, and normalized to unity at the isocenter.

Central axis depth dose (CADD) was measured with the same water tank with A1SL ion chamber for 5 cm × 40 cm field size (all leaves open) up to the depth of 20 cm. The depth of dose maximum (Dmax) was estimated from these measurements. For output measurements, same A1SL ion chamber was placed in a stack of 15 cm × 55 cm Virtual Water phantom (density 1.04 gm/cc) slabs (Med-Cal, Verona, WI) for a field size of 5 cm × 40 cm at an isocenter (85 cm) with buildup of 1.5 cm. The charge collected by the ion chamber was recorded by an eight channel TomoElectrometer. This charge was then corrected for the output estimation. Though the dosimetry protocol (AAPM TG 51) is not truly valid for Tomotherapy, the calibration factor traceable to reference standard was used and the output was estimated with KQ as 0.9965.

To further validate the dosimetry performed with a dedicated A1SL ion chamber and Tomoelectrometer, an independent dosimetry system was used. A cylindrical ion chamber FC65G (0.065 cc, Scanditronix Welhoffer, Sweden) was used with DOSE1 electrometer (Scanditronix Welhoffer, Sweden) to measure the output. The ion chamber was placed at 1.5 cm in the virtual water slabs at SAD 85 cm. A similar static 60 seconds procedure was performed for the field size of 5 cm × 40 cm. The readings recorded by the electrometer were converted to the dose rate (output) and then compared with that measured with A1SL ion chamber.

CADD was measured up to 12.5 cm depth with a parallel plate ion chamber (0.05 cc, PPC05, Scanditronix Welhoffer, Sweden). In both the measurements, a backscatter of 10 cm was used. The readings from PPC05 ion chamber were normalized to the depth of dose maximum (Dmax) and then the percentage CADD was determined.

The subsequent CADD measurements were also carried out with thermoluminescence dosimeter (TLD). The TLDs in powder form [(TLD 100), (LiF:Mg, Ti)] was used. Prior to each irradiation, TLD powder (The Harshaw Chemical Co. Solon, Ohio, USA) were annealed using a thermal cycle: 400°C (plus/minus 5°) for one hour-cooling for five minutes −100°C for two hours in a Programmable Muffle Furnace (Model-126, Fisher Scientific Co. Pittsburgh, PA USA) and then cooled to normal room temperature. For annealing, the TL powder was placed inside a glass Petri dish with cover. Rexon UL-320 TLD Reader, (TLD systems Inc. USA) was used to record TL output at maximum acquisition temperature of 280° C using constant heating rate of 14°C/sec. A constant time gap of 24 hours was maintained between irradiation and read out. Dose response curve for the TLD-100 powder was generated in Co-60gamma ray beam (Equinox 80, Best Medical Canada) and was found linear in the range of 0.5- 4.0 Gy. For measurements using TLD, about 40mg of the freshly annealed TLD-100 powder was packed in square polyethylene pouch (approximately 1cm × 1cm). The TL output, of about 10mg powder, was recorded using Rexon TLD reader and this way four readings were obtained from each TL pouch. The mean value of net TL output per unit weight (nC/mg) of these four readings was used in calculation. The uncertainty in TLD-100 powder measurements was ±2%.

The TLD packet was placed at the surface and at various levels of depth (from 1 to 10 cm) in central axis at SAD of 85 cm in virtual water slabs. The field size of 5 cm × 40 cm was selected for these measurements. The same 60-second procedure with static gantry and couch was performed. The CADD was measured up to the depth of 17 cm. The TLDs were evaluated using a commercial TLD-reader system (REXON Model UL-320 reader, Ohio, USA) after 24 h and the average of the readings was estimated. The surface dose was estimated from these readings by taking the ratio of surface dose reading to the reading at the depth of dose maximum (Dmax). The linac and the target were replaced after eight months since commissioning and the same measurements were repeated and compared.

## Results

The majority of the data was analyzed with the dedicated software programs available only with the vendor. The results of MLC TandG procedure revealed acceptable linac X axis alignment. The MLC is positioned with respect to centre of rotation and is aligned with respect to the radiation plane. Percent out of focus was found to be 0.38 (specification less than 2%). The linac shift was found to be 0.026 mm (spec less than 0.3 mm). This test reveals that the source was 0.026 mm away from the center of the primary collimator, after accounting for the magnification of the movement at the isocenter. Hence, no movement of the linac in X-direction was recommended.

The measured data was in good agreement with the reference data. The results showed an acceptable tilt of one to two per cent. The energy constancy was found to be 99% (range 99-100.5) with maximum gamma as 0.4.

**Figure 3:**
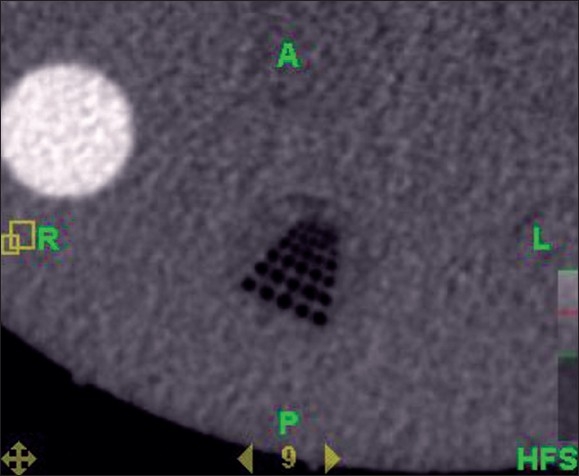
At least three rows of the resolution plugs are clearly visible. In addition, all inserted density plugs are visible from the registration panel. Moreover, the plugs can be individually resolved. The image was found to be free of rings and streak artifacts.

The quality of TomoImage can affect registration results. The MVCT checks confirmed that the image could be reconstructed in the registration panel. The measured dose during MVCT procedure (seven slices) was found to be 2.57 cGy. Figure 3 shows that at least three rows of the resolution plugs are clearly visible. In addition, all inserted density plugs are visible from the registration panel. Moreover, the plugs can be individually resolved. The image was found to be free of rings and streak artifacts. The plug diameters were in well agreement with the actual one. All these above MVCT tests were found to be passed.

**Figure 4:**
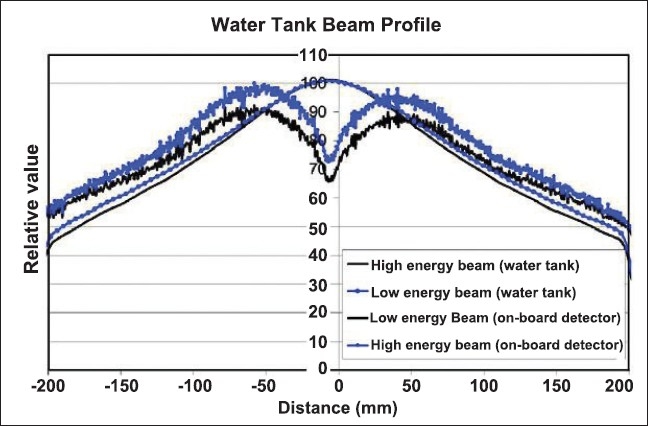
The resultant dose profiles (lateral) measured with the ion chamber. The high energy and low-energy beam lateral beam profiles were first measured in water at a depth of 1.5 cm using an A1SL ion chamber. The SSD was 85 cm. The resultant dose profiles (lateral) measured with the on board MVCT detector. The same high-energy and low-energy beams were measured. The couch was positioned such that nothing attenuated the beam except the gantry covers.

**Figure 5:**
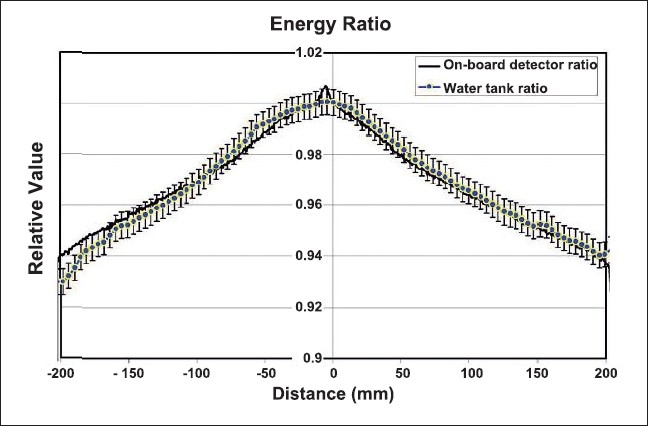
The water-tank-measured, high-energy beam profile was divided by the water tank measured, low-energy beam profile and normalized to unity at the isocenter. Likewise, the onboard detector measured highenergy profile was divided by the low-energy profile, and normalized to unity at the isocenter. Both plots are shown. Vertical error bars of +/− 0.5% were added to the water tank measured normalized plot.

The resultant dose profiles for high-energy and low-energy beams measured with ion chamber and MVCT detectors are shown in Figure 4. Both plots are shown in Figure 5. Vertical error bars of +/− 0.5% were added to the water tank measured normalized plot. There is excellent agreement between the two normalized plots except at negative 18 to 20 cm on one side of the plot, and there is a slight anomaly at the center of the detector-normalized plot.

The ratio of reading at 20 cm to 10 cm is defined as the ionization ratio in AAPM TG-21, which is related to the nominal accelerating potential (energy) of the x-ray source. The reference ratio for energy was 0.52 while the mean ratio was 0.515. The ratio 0.52 was for 6 MV energy used for the treatment. The variation between the mean ratio and the reference ratio was found to be −0.96%. The reference ratio for energy was in line with recommended beam quality by international protocols (AAPM TG-51 and IAEA TRS-398, etc.). This result revealed the energy to be constant within +/− 1.5% over a period of twelve months when measured with A1SL ion chamber in a water tank.

The output (885 cGy/min) measured with A1SL ion chamber in virtual water showed mean variation of 0.6% compared to the nominal dose rate (890 cGy/min). This output was consistent within +/− 2% for a period of 12 months. Similarly, the output measured with FC65G ion chamber (875 cGy/min) revealed a mean variation of 1.1% and 1.7% with the A1Sl ion chamber and reference output respectively. The dose delivered to TLD at 1.5 cm depth in virtual water slabs was estimated to be 878 cGy/min and was in very good agreement (0.8%) with the nominal dose rate of the machine measured with the A1SL ion chamber. The output was consistent even after the major repairs.

**Figure 6:**
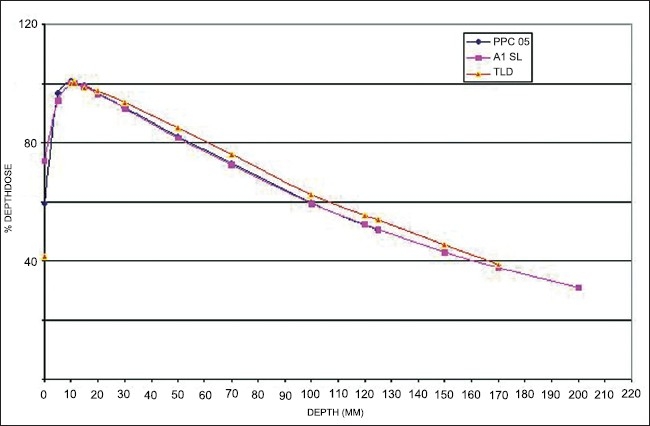
Comparison of CADD measured with PPC05 parallel plate ion chamber, A1SL ion chamber and TLD.

Figure 6 shows the comparison of CADD measured with PPC05 parallel plate ion chamber, A1SL ion chamber and TLD. The data shows good agreement with each other. The CADD values measured with parallel plate ion chamber were within +/− 2% when compared with water tank measurements. From the average sampling data of the TLDs, the estimated surface dose for 6 MV photon beam from a Tomotherapy machine with static measurements was 40%.

## Discussion

The fine resolution of the detectors offers two benefits for IGT: the obvious one being the acquisition of volumetric CT imaging of the patient at the time of treatment delivery. Secondly, during treatment, the intensity of the photons that exit the patient can be collected and used in a back-projection computation to assess the dose distribution that is delivered to the patient. The imaging beams are produced at a lower quality/ energy (3.5 MV) than the treatment beams and the output of the guide is reduced. This results in the acquisition of volumetric images at very acceptable doses, typically between 0.5 cGy and 3 cGy, which are comparable with doses required to obtain planar images on contemporary MV electronic portal imaging devices (EPIDs). We measured the dose during MV imaging and found to be 2 cGy for seven slices. This was well within the acceptable tolerance (less than 4 cGy) from the manufacturere. The nominal energy of x-ray beams used for imaging and treatment was 3.17 MV and 6 MV respectively.

The detectors have thin tungsten septa that separate the ionization chambers; a significant number of electrons released at photon scattering events in the septa are conducted into the gas chambers and detected. So, whereas the septa were included to reduce cross-talk “noise” between detectors for kV imaging, they result in an increase in efficiency for MVCT imaging. Gas leakage was observed incidentally and the MVCT detectors were replaced with a new one. All the tests were repeated and the performance of the detectors was evaluated and compared with the old one. The performance of the new MVCT detector was comparable with the earlier data and thus was acceptable.

Tomotherapy has written software for facilitating the analysis of film and detector data. Any test that can be performed with the detector data can be automated and analyzed quickly. The detector array proved very useful for analysis. It was able to analyze its consistent mapping with the MLC leaves and its alignment with the gantry rotation plane. It was found that it is capable of adequately replacing film for few tests. The spatial resolution of the detectors was almost as fine (0.6 mm) as digitized film results, as determined by the transverse profiles.

The TandG profile can be obtained by film, or by scanning an ion chamber across the all leaves open, odd leaves open, and even leaves open profiles. However,, it is easier and faster to utilize the onboard MVCT detector to collect the profiles. We used this MVCT detector data. The data were processed such that the all-even-leaf profile was added to all-odd-leaf profile. This determined how well the xenon detectors could differentiate the fluence.

The absence of flattening filter in the tomotherapy machine lowers the head scatter contribution which is not the case in conventional linacs. Hence, the shape of the transverse profile is not flat as compared to the conventional linac. This profile is modulated by the MLC.

The rotational variation test results showed that the machine is stable with rotation and the subsequent constancy in the output and energy was observed. The energy of the beam changes the shape of the profile of linac. Higher energy beams are more forward directed and have lowered shoulders with respect to the center. Placing the MVCT detector array in a position such that it is aligned to the center of the jaws ensures that the optimum detector response will be obtained during a TomoImage scan, maximizing image quality with regard to noise.

This study aimed at the consistency of static output measurement for first Tomotherapy machine installed at our centre and its verification by an independent dosimetry system. Output and the energy are daily measured to monitor the beam stability for the static treatment. Though IMRT treatments in Tomotherapy are not of static type, this method gives the quick check to monitor the dose rate of the machine. However, we do measure the output for rotating gantry/fixed couch with the Xenon detectors. The data is analyzed and the output monitored. Since this study focuses on the measurements with the ion chamber and keeping in mind the AAPM TG-51, the detector analysis may divert our primary aim. None of the dosimetry protocols are directly applicable in Tomotherapy. However, the calibration factor traceability should be valid. Our results were in good agreement with the earlier reports.[7]

Secondly, as mentioned earlier, unlike linear accelerator, Tomotherapy does not strictly follow the concept of monitor units (MU) and output as mentioned in dose rate (either cGy/min or Mu/Min). We used cGy/Min for reporting the output of Tomotherapy.

Measuring system during commissioning of the tomotherapy machine was virtual water only and placing three ion chambers one above the other at different depths does not exhibit any dose perturbation. The detector response was consistent for the period of twelve months. Thus the variation of beam quality over a period of 12 months was negligible.

Overall, from physicist's point of view, the daily quick check of output and energy is mandatory and this does not take more than five minutes. It should be performed on a daily basis to continuously monitor the beam stability in a Tomotherapy machine where one needs to adopt the technology. Thus, the intent should be for the technology transfer as well.

This paper deals with the validation of the Tomotherapy dosimetry with an independent dosimetry system. The tests performed in this study were part of quality control. We used parallel plate ion chamber and TLD for this purpose. Both of these dosimeters are the benchmark dosimeters for output and CADD measurements. The TL material used for in-vivo dosimetry include: lithium fluoride (LiF-100), lithium borate (Li_2_ B_4_ O_7_), and calcium sulphate (CaSo_4_), TL detectors either in the form of powders or rods, chips or pellets. Their small dependence on dose rate, temperature, and energy in the therapeutic range and their wide applicable dose range make TLDs suitable for in-vivo dosimetry. The TLD to be used for patient dosimetry is annealed in an oven 24 hrs prior to use.

The TLD being in powder form does not need specific batching and thus reduces the uncertainty in measurement. However, the uncertainty in the TLD measurements was +/−3%. The parallel plate ion chamber and the electrometer has been calibrated from the national standard dosimetry laboratory, Bhabha Atomic Research Centre (BARC, Mumbai). Extreme precaution was taken while handling the TLDs.

The Tomotherapy machine at our centre was clinically commissioned in September 2007 and more than 100 patients have been treated so far. As part of our quality assurance protocol, we measure the output and energy on a daily basis. The Tomotherapy dosimetric accuracy was excellent after major repairs as well and does not affect clinical treatment. The results from the dosimeteric study were encouraging and thus it assured that the Tomotherapy delivers a safe dose for improving the quality of the treatment. The output measured here was for only one field size 5 cm × 40 cm. This was to simulate the reference equivalent field size of 10 cm × 10 cm as the case in routine dosimetry protocols and hence the measurement for various jaw settings was not performed.

CADD measurement with parallel plate ion chamber was performed till 12.5 cm only. We could not lower the couch further with minimum backscatter of 10 cm. However, the CADD values were in good agreement with the A1SL ion chamber till 10 cm depth. Similar was the case for TLDs. CADD was measured up to 17 cm depth with TLDs. Further depth was not possible due to the technical problem of lowering the couch with the sufficient backscatter.

The surface dose is an important issue in radiotherapy. Hence, it was attempted to measure the surface dose with TLD for a static Tomotherapy procedure. The estimated surface dose was relatively on a higher side. This is because the isocenter is close to the source (85 cm) which is not the case in linear accelerator where the isocentre is at 100 cm. The measurements performed with TLDs were reasonably comparable with the ion chamber measurements.

## Conclusion

This agreement indicates that the on-board detector can be used to measure the consistency of the lateral beam profile shape. The spatial resolution of the detectors was almost as fine as digitized film results. Any test that can be done via the xenon detectors can be automated and the results can be known in nearly real time. The dosimetric stability of Tomotherapy system was excellent for the period of 12 months since commissioning and even after the major repairs. Both the output and energy were consistent and within the acceptable limits. The measurements carried out with independent dosimeter (FC 65G cylindrical ion chamber, parallel plate ion chamber and DOSE1 electrometer) were satisfactory and acceptable. The TLDs measurements supported these measurements as well. Thus, it is recommended to use an independent dosimeter for Tomotherapy dosimetry to validate their dosimeters.
